# Inflammatory biomarkers do not distinguish between patients with sciatica and referred leg pain within a primary care population: results from a nested study within the ATLAS cohort

**DOI:** 10.1186/s12891-019-2604-2

**Published:** 2019-05-10

**Authors:** Samantha L. Hider, Kika Konstantinou, Elaine M. Hay, John Glossop, Derek L. Mattey

**Affiliations:** 1 Arthritis Research UK Primary Care Centre, Research Institute of Primary Care and Health Sciences, Keele University, Keele, Staffordshire ST5 5BG UK; 2 Midlands Partnership Foundation Trust, Haywood Hospital, Stoke-on-Trent, Staffordshire ST6 7AG UK

**Keywords:** Sciatica, Low back pain, Cytokines, TNFα, Radiculopathy

## Abstract

**Background:**

There is increasing interest in the role of pro-inflammatory cytokines in the pathogenesis of sciatica and whether these could be potential targets for treatment. We sought to investigate serum biomarker levels in patients with low back-related leg pain, including sciatica.

**Methods:**

Primary care consulters aged > 18 with low back-related leg pain were recruited to a cohort study (ATLAS). Participants underwent a standardised clinical assessment, lumbar spine MRI and a subsample (*n* = 119) had samples taken for biomarker analysis. Participants were classified having: a) clinically confirmed sciatica or referred leg pain, and then subdivided into those with (or without) MRI confirmed nerve root compression due to disc prolapse. Seventeen key cytokines, chemokines and matrix metalloproteinases (MMPs) implicated in sciatica pathogenesis including TNFα and IL-6, were assayed in duplicate using commercial multiplex detection kits and measured using a Luminex suspension array system. Median biomarker levels were compared between the groups using a Mann Whitney U test. Multivariate logistic regression analysis was used to investigate the association between clinical measures and biomarker levels adjusted for possible confounders such as age, sex, and symptom duration.

**Results:**

No difference was found in the serum level of any of the 17 biomarkers tested in patients with (*n* = 93) or without (*n* = 26) clinically confirmed sciatica, nor between those with (*n* = 44) or without (*n* = 49) sciatica and MRI confirmed nerve root compression.

**Conclusion:**

In this cohort, no significant differences in serum levels of TNFα, IL-6 or any other biomarkers were seen between patients with sciatica and those with back pain with referred leg pain. These results suggest that in patients with low back-related leg pain, serum markers associated with inflammation do not discriminate between patients with or without clinically confirmed sciatica or between those with or without evidence of nerve root compression on MRI.

## Background

Lumbar spinal radicular pain (with or without radiculopathy) or sciatica, represents one distinct presentation of low back-related leg pain, which is often characterised by pain radiating to below the knee and into the foot. Patients with sciatica suffer more severe pain and disability and take longer to recover than those with low back pain (LBP) alone [1]. Although traditionally initially thought to be caused by mechanical nerve root compression, usually by a herniated disc, studies suggest inflammation (mediated by cytokines) also contributes to the pathogenesis of sciatica [2].

Interest on possible inflammatory aetiologies for sciatica initially focused on the cytokine TNFα, including randomised trials of anti-TNF inhibitors infliximab [3] and adalumimab [4], showing some improvement in leg pain and reduction in numbers of patients needing spinal surgery [4], with meta-analyses suggesting further trials are warranted [5–7]. These therapies may represent a novel way to treat sciatica but are expensive, and given that sciatica is common, data is required to effectively target these drugs at patients more likely to derive benefit.

Other pro-inflammatory cytokines investigated include IL-6- with studies showing high levels of IL-6 in disc samples from patients undergoing surgery [8]. A study by Pederson et al. [9] showed higher serum levels of IL-6 and IL-8 in patients with persistent sciatic pain, defined as pain levels on a Visual Analogue Score (VAS) of > 3, compared to those with pain levels on VAS of < 3 at 12 months. A second study by Wang et al. [10] also showed higher levels of IL-6, IL-8, TNFα and IL-4 in patients with severe sciatica (defined as pain VAS > 3) compared to those with mild sciatica (VAS < 3) or healthy controls. However patients with severe sciatica had a long duration of symptoms (mean (SD) duration 48 (28.96) weeks) and were recruited from a secondary care spinal clinic and thus it is not clear whether these elevated biomarker levels represent patients with chronic or severe symptoms or whether these biomarker levels are elevated in a less selected population. Such an approach would help further elucidate whether these biomarkers are elevated early in the symptom course or could be used in predicting outcome or guide treatment for sciatic pain.

Therefore, the aim of this study was to examine serum levels of key biomarkers in patients presenting in primary care with symptoms of low back-related leg pain including sciatica, and to see whether levels were different in patients with clinically diagnosed sciatica with or without or evidence of nerve root compression on MRI.

## Methods

### Participants

Primary care consulters aged > 18 with low back-related leg pain were recruited as part of a longitudinal study (the ATLAS study) investigating the overall prognosis of low back-related leg pain and sciatica in primary care. The ATLAS study procedures have been described in detail elsewhere [11], here we give brief information on recruitment and assessment details. Patients consulting their GP (General Practitioner) low back and leg pain, who were potentially eligible for the ATLAS study, were sent a letter with information about the study, an invitation to attend the research clinic, and baseline questionnaires capturing sociodemographic, pain, psychological and health variables. Patients with other inflammatory conditions, such as rheumatoid arthritis were excluded. At the research clinic, patients underwent a standardised clinical assessment by one of seven experienced musculoskeletal physiotherapists, and were diagnosed as having clinically defined sciatica (spinal nerve root involvement) or referred (non-specific) leg pain, based on the examiner’s clinical opinion. Providing there were no clinical contraindications to the procedure, patients had a lumbar spine magnetic resonance imaging (MRI) scan within 2 weeks of their baseline assessment. The MRI findings were not part of the clinical diagnosis of sciatica or referred leg pain. All MRIs were scored by a single assessor, a senior consultant musculoskeletal radiologist. The assessor provided a clinical report indicating the presence/absence of definite or possible nerve root compression by lumbar spinal level (3 lower lumbar levels) and side (right/left) and the reason(s) for it if present (e.g. disc prolapse, stenosis) as per normal practice in radiological reporting.

For the purposes of this nested study (which for practical considerations, i.e. availability of phlebotomy service and storage for blood samples, could only be conducted at a single research site), patients consenting to the main study at this site, were also invited to take part in the biomarker sub study. Consenting patients had serum samples taken for biomarker analysis in addition to the standardised clinical assessment.

Participants were classified as having nerve root involvement due to suspected disc prolapse on the basis of the clinical assessment (clinically defined: yes/no).

For the purposes of analysis, we first examined levels of serum biomarkers between patients with clinically diagnosed disc-related sciatica and with referred leg pain, and secondly we examined levels of serum biomarkers between patients with clinically diagnosed disc-related nerve root involvement and evidence of concordant MRI findings, and those patients without evidence of concordant MRI findings.

All patients provided written informed consent. The biomarker study was approved by the North West- Greater Manchester South Research Ethics Committee (REC reference number: 12/NW/0173).

### Measurement of cytokines, chemokines and MMPs

Sera were separated from bloods collected in plain BD Vacutainer® tubes at study entry. All sera were stored at -70^o^ C until required. Key cytokines, chemokines and matrix metalloproteinases (MMPs) implicated in sciatica pathogenesis including TNFα, IL-1, IL-6, MMP1,3,8, aggregan and monocyte chemoattractant protein-1 (MCP-1/CCL-2) were measured in duplicate on a Bio-Plex™ 200 suspension array system using commercially available multiplex detection kits for cytokines (Milliplex multi-analyte profiling (MAP) kits, Millipore (UK) Ltd., Walford, Hertfordshire, UK), or Fluorokine MAP kits for MMPs (R&D Systems Europe, Abingdon, Oxfordshire, UK). Further details on the kits used are available in the Appendix. All assays were carried out according to the manufacturers’ instructions. High and low control samples were used in each assay.

### Statistical analysis

All data were tested for normality and the appropriate parametric or non-parametric test was selected for analysis. Initial univariable analysis was carried out using the Mann Whitney U test to compare median cytokine levels between groups. A multivariate variable selection procedure using the algorithm of McHenry [12] was used to select biomarker variables which showed the strongest association with either clinically determined sciatica, with/without MRI evidence of nerve root compression. Multivariate logistic regression analysis was used to investigate the association between clinical measures and biomarker levels while adjusting for possible confounders such as age, sex, and symptom duration.

Statistical analyses were carried out using Number Cruncher Statistical Software package for Windows (NCSS 2000, NCSS Statistical Software, Kaysville, Utah, USA). The significance level was set at a *p* value of 0.05.

## Results

Data for the main ATLAS cohort are published elsewhere [13]. One hundred nineteen patients were recruited to the biomarker sub study, of these 73 (61.2%) were female. Ninety three (78%) were clinically diagnosed with sciatica and of these, 44 (47.3%) had MRI confirmed nerve root compression due to disc prolapse. Table 1 shows the baseline demographics of the cohort.

**Table 1:** Comparison of key characteristics for patients clinically diagnosed with or without sciatica. All figures are frequencies (percentages) unless stated otherwise as mean (SD)

Characteristics	Clinically defined sciatica *n* = 93		Referred pain *n* = 26
	MRI + ve (*n* = 44)	MRI –ve (*n* = 49)	
Socio-demographics			
Age (years), mean (SD)	55.2 (11.7)	50.3 (14.7)	49.5 (11.1)
Female gender, *N* (%)	24 (55)	32 (65)	17 (65)
BMI, mean (SD)	30.8 (6.3)	29.5 (6.2)	32.5 (8.2)
Current smoker, *N* (%)	17 (39)	14 (28)	9 (34)
Pain			
RMDQ disability score (0–23), mean (SD)	12.5 (5.1)	11.8 (6.0)	11.8 (6.8)
Back pain intensity NRS, mean (SD)	6.6 (2.3)	6.7 (2.2)	6.9 (2.3)
Leg pain intensity, NRS, mean (SD)	6.4 (2.4)	6.1 (2.4)	7.0 (2.4)
Pain below knee, *N* (%)	30 (71)	28 (61)	21 (84)
Leg pain is worse, *N* (%)	21 (49)	22 (45)	16 (61)
Duration of current symptoms^a^			
Back pain			
Back pain< 6 weeks, *N* (%)	15 (36)	14 (29)	7 (27)
Back pain 6–12 weeks, *N* (%)	12 (29)	8 (17)	7 (27)
Back pain > 3 months, *N* (%)	15 (36)	26 (54)	12 (46)
Leg pain			
Leg pain< 6 weeks, *N* (%)	17 (40)	15 (33)	8 (32)
Leg pain 6–12 weeks, *N* (%)	15 (36)	9 (20)	8 (32)
Leg pain> 3 months, *N* (%)	10 (24)	22 (48)	9 (36)

^a^There was a small proportion of missing data for symptom duration

None of the biomarkers tested showed any difference in level between patients with or without clinically confirmed sciatica, nor did they show any differences between sciatica patients with MRI findings of nerve root compression (*n* = 44) and those without (*n* = 49). Serum IL-6 levels were low or below the level of detection, and no difference was seen in the levels of IL-6 or TNFα or any of the other biomarkers between the groups (Table 2). None of the biomarkers selected were significantly associated with either clinically determined sciatica or sciatica with positive MRI findings, in the multivariate variable selection procedure or logistic regression analysis. There was no evidence of significant multicollinearity.

**Table 2:** Median biomarker levels in those with clinically defined sciatica and in those with and without MRI findings

Biomarker	Clinically defined sciatica		Clinically defined sciatica plus MRI changes^a^	
	No (*n* = 26)	Yes (*n* = 93)	No (*n* = 49)	Yes (*n* = 44)
TNFα	11.9 (9.7–13.8)	10.7 (1.0–14.8)	12.39 (1.0–14.74)	9.86 (1.0–14.96)
IL-1β	BLD	BLD	BLD	BLD
IL-6	BLD	BLD	4.96 (1.6–7.4)	4.21 (0.5–7.7)
IL-33	9.0 (7.2–10.9)	8.0 (6.9–10.4)	9.1(7.9–10.1)	8.8 (7.3–10.1)
IFNγ	BLD	BLD	BLD	BLD
TNFRI	5117.1	5087.4	5059.9	5190.3
	(4379.9–5692.6)	(4525.4–6033.3)	(4660.4–5838.9)	(4418.2–6113.9)
IL-1RI	1488.8	1469.6	1490.5	1423.9
	(1311.8–1620.9)	(1273.0–1626.3)	(1346.4–1615.8)	(1143.8–1654.0)
IL-1RII	6913.9	6985.1	7347.6	6797.3
	(5541.3–7819.7)	(6099.9–8066.3)	(6513.9–8414.0)	(5818.4–7825.0)
IL-1Ra	866.3	998.4	974.4	1019.7
	(575.8–1267.8)	(735.8–1421.6)	(693.9–1419.7)	(7417.7–1508.9)
CXCL8	2.8	3.6	3.7	4.7
	(0.9–3.5)	(1.5–6.4)	(1.7–6.5)	(2.3–7.1)
MCP-1	313.7	257.9	274.3	310.0
	(247.7–313.7)	(207.8–361.8)	(191.1–348.2)	(213.6–416.0)
FGFβ	BLD	BLD	BLD	BLD
VEGF	42.9 (19.4–62.2)	65.0 (22.0–98.5)	66.2 (27.6–96.9)	63.5 (20.7–123.8)
HGF	265.5	245.9	244.9	245.9
	(212.5–351.9)	(191.0–321.7)	(182.6–315.1)	(196.9–315.1)
Ang-2	2164.8	2465.0	2311.2	2870.0
	(1637.7–3385.0)	(1842.1–3375.4)	(1659.9–3070.6)	(1921.6–3456.1)
MMP-1	8450.0	7857.2	7344.9	8011.0
	(4267.3–13,173.5)	(5031.0–15,134.3)	(5113.1–12,124.3)	(4171.7–24,762.6)
MMP-2	46,778.4	44,214.2	42,714.7	44,418.4
	(45,258.0–48,596.9)	(37,585.8–50,496.4)	(36,594.9–50,419.9)	(38,275.4–50,668.8)
MMP-3	13,741.5	12,111.6	12,852.8	11,882.8
	(8626.9–22,480.1)	(8403.3–18,651.9)	(7733.5–18,337.4)	(8863.1–18,789.5)
MMP-8	8942.1	7409.5	7842.8	6091.0
	(4098.4–11,552.5)	(4509.8–12,196.8)	(4518.1–10,694.5)	(4181.4–13,098.2)
Aggrecan	943.0	922.9	911.2	961.4
	(747.4–1302.5)	(618.8–1253.4)	(624.7–1272.3)	(589.5–1243.3)

*BLD* majority of samples below the limit of detection

^a^MRI findings of nerve root compression in patients with clinically defined sciatica. (i.e. changes Y/N). Values shown are the median (interquartile range) in pg/ml

## Discussion

LBP and sciatica are common health problems with significant impact at both the individual level (in terms of pain and disability) and the health and societal level (in terms of healthcare costs and work loss). Studies have suggested a possible inflammatory aetiology in the development or persistence of sciatic pain, and given the availability of cytokine inhibitors for treatment of inflammatory arthritis, this has prompted research to determine whether these agents could be effective for sciatica. Previous studies have confirmed the presence of key cytokines such as TNFα and IL-6 in surgical disc samples, and recent data have suggested that serum levels of these cytokines may be elevated in patients with sciatica symptoms [8–10], although other studies have shown no association [14].

In this study of patients with low back-related leg pain seeking primary healthcare, no difference was found in the levels of TNFα or IL-6 between those with or without either clinically defined sciatica or clinically defined sciatica with concordant MRI findings. The results of this study are in contrast to those of Wang [10] who found elevated levels of TNFα and IL-6 in patients with severe sciatica (defined as pain level of > 3 on VAS). However, in part this difference may be explained by the characteristics of the patients recruited in the study by Wang et al. [10], as patients were recruited from a spinal clinic and had a mean symptom duration of 48 weeks. By contrast, our cohort was recruited from primary care attenders who had a shorter duration of symptoms, with over one third of patients having leg pain duration of less than 6 weeks.

There are a number of strengths and weaknesses that need to be considered when interpreting the results of this study. A key strength is that the cohort represents an unselected population of primary care consulters with low back-related leg pain and sciatica rather than being recruited from spinal surgical clinics and as such the results are likely to be highly generalizable. Furthermore, patients underwent a standardised clinical assessment and MRI scan [11], providing confidence in the likely diagnosis of radicular pain. As all participants in the biomarker sub-study were recruited from a single research clinic, all samples were taken at a similar time of day thus reducing biological variability.

One of the weaknesses is that biomarker measurements were made at a single time point only, so we are not able to determine whether there were fluctuations in the levels of biomarkers over time (associated with the level of pain for example. Other hypotheses that may explain our negative results are that firstly we did not have a group of pain free controls for comparison (albeit that this was not the original aim of the study) or that the groups were not sufficiently different to detect differences. Since there are currently no internationally agreed standard benchmark serum levels for any of these biomarkers, comparisons across studies is not possible [15]. Secondly, given that some of the subgroups were small the study was underpowered to be able to detect differences in biomarker levels between the subgroups. Nevertheless, the unselected primary care population, who underwent standardised assessments, ensures that the cohort is generalizable. Further longitudinal studies are required to determine whether cytokine levels become elevated in those with persistent symptoms rather than those with a short symptom duration.

## Conclusion

In summary, in our primary care cohort of patients with low back-related leg pain including sciatica, serum markers associated with inflammation, angiogenesis or extracellular matrix remodelling do not discriminate between patients with and without sciatica, or between those with sciatica with and without obvious evidence of nerve root compression on MRI. Larger longitudinal studies are required to determine whether any serum biomarker levels are associated with persistence of sciatica symptoms or whether other biomarkers could be important in the pathogenesis and persistence of sciatica.

## Appendix

The Milliplex-MAP cytokine assay from Millipore UK was a custom designed assay based on product HCYTOMAG-60 K which allows the choice of up to 60 different cytokines. The MMP assay from R&D Systems was also custom designed using a Fluorokine MAP Base kit for MMPs (cat no LMP000) and individual MMP assays (MMP-1 cat no LMP091, MMP-2 cat no LMP902, MMP-3 cat no LMP513, MMP-8 cat no LMP908).

## Abbreviations

GP: General Practitioner
IFN: Interleukin
IL: Interleukin
LBP: Low back pain
MCP: Monocyte chemoattractant protein
MMP: Matrix Metalloproteinase
MRI: Magnetic resonance imaging
REC: Research Ethics Committee
SD: Standard deviation
TNF: Tumour necrosis factor
VAS: Visual Analogue Score
